# Exonic Re-Sequencing of the Chromosome 2q24.3 Parkinson’s Disease Locus

**DOI:** 10.1371/journal.pone.0128586

**Published:** 2015-06-19

**Authors:** Catherine Labbé, Kotaro Ogaki, Oswaldo Lorenzo-Betancor, Minerva M. Carrasquillo, Michael G. Heckman, Allan McCarthy, Alexandra I. Soto-Ortolaza, Ronald L. Walton, Timothy Lynch, Joanna Siuda, Grzegorz Opala, Anna Krygowska-Wajs, Maria Barcikowska, Krzysztof Czyzewski, Dennis W. Dickson, Ryan J. Uitti, Zbigniew K. Wszolek, Owen A. Ross

**Affiliations:** 1 Department of Neuroscience, Mayo Clinic, Jacksonville, Florida, United States of America; 2 Section of Biostatistics, Mayo Clinic, Jacksonville, Florida, United States of America; 3 Dublin Neurological Institute at the Mater Misericordiae University Hospital, Conway Institute of Biomolecular & Biomedical Research, University College Dublin, Dublin, Ireland; 4 Department of Neurology, Medical University of Silesia, Katowice, Poland; 5 Department of Neurology, Jagiellonian University, Krakow, Poland; 6 Department of Neurodegenerative Disorders, Medical Research Centre, Polish Academy of Sciences, Warsaw, Poland; 7 Department of Neurology, Central Hospital of the Ministry of Interior and Administration, Warsaw, Poland; 8 Department of Neurology, Mayo Clinic, Jacksonville, Florida, United States of America; 9 Department of Pathology, Mayo Clinic, Jacksonville, Florida, United States of America; Harvard Medical School, UNITED STATES

## Abstract

Genome-wide association studies (GWAS) in Parkinson’s disease (PD) have identified over 20 genomic regions associated with disease risk. Many of these loci include several candidate genes making it difficult to pinpoint the causal gene. The locus on chromosome 2q24.3 encompasses three genes: *B3GALT1*, *STK39*, and *CERS6*. In order to identify if the causal variants are simple missense changes, we sequenced all 31 exons of these three genes in 187 patients with PD. We identified 13 exonic variants including four non-synonymous and three insertion/deletion variants (indels). These non-synonymous variants and rs2102808, the GWAS tag SNP, were genotyped in three independent series consisting of a total of 1976 patients and 1596 controls. Our results show that the seven identified 2q24.3 coding variants are not independently responsible for the GWAS association signal at the locus; however, there is a haplotype, which contains both rs2102808 and a *STK39* exon 1 6bp indel variant, that is significantly associated with PD risk (Odds Ratio [OR] = 1.35, 95% CI: 1.11–1.64, P = 0.003). This haplotype is more associated than each of the two variants independently (OR = 1.23, P = 0.005 and 1.10, P = 0.10, respectively). Our findings suggest that the risk variant is likely located in a non-coding region. Additional sequencing of the locus including promoter and regulatory regions will be needed to pinpoint the association at this locus that leads to an increased risk to PD.

## Introduction

Parkinson’s disease (PD) was not historically considered a genetic disease until in depth studies of the segregation of genetic variants in families revealed several inherited mutations in genes such as *SNCA*, *LRRK2*, and *PARK2*[1, 2]. These first discoveries were followed by population based genome-wide association studies (GWAS) aimed at identifying risk factors for sporadic PD, which represents up to 90% of PD cases[3]. To date, GWAS have nominated over 20 loci influencing the risk to PD[4]. Causal genes have been nominated for a few of the loci (mostly because they overlap with familial PD genes) but the majority of GWAS loci are defined by large regions of linkage disequilibrium (LD) containing several different genes.

The chromosome 2q24.3 locus was associated with increased PD risk in 2011 through a meta-analysis of GWAS published by Nalls *et al*[5] and has since been replicated in even larger meta-analytical approaches [6, 7]. There has also been independent replication in Caucasian populations although the association has not been observed in Asian series of Han Chinese descent [8–11]. A recent GWAS in Ashkenazi Jewish patients also identified an association signal in the region, although the sample size was limited and significance was not achieved [12]. Serine Threonine Kinase 39 (*STK39*) has been put forward as the causal gene, but the locus, as defined by Nalls *et al*, contains two other candidate genes (UDP-Gal:BetaGlcNAc Beta 1,3-Galactosyltransferase, Polypeptide 1 [*B3GALT1*] and Ceramide Synthase 6 [*CERS6*]). In order to identify the potential causal variant(s) responsible for the GWAS signal, the region needs to be re-sequenced and fine-mapped.

Variants in coding regions are likely to have an effect on protein structure and function and thus a great impact on phenotype. Therefore, in the present study we undertook the screening of all 31 exons of genes *B3GALT1*, *STK39* and *CERS6* in 187 patients with PD. We identified 13 exonic variants including four non-synonymous and three insertion/deletion variants. After validation in controls, we genotyped the seven non-synonymous and insertion deletion variants in three independent series (US, Irish, and Polish) consisting of a total of 1976 patients and 1596 controls. We did not identify a single variant responsible for the risk at the 2q24.3 locus but we observed a haplotype that included a *STK39* coding variant which was significantly associated with PD risk.

## Materials and Methods

### Study subjects

A total of 1976 patients with clinically diagnosed PD and 1596 controls were included in this case-control study. The patients are all unrelated non-Hispanic Caucasians of European descent. Subjects were from a US series collected at Mayo Clinic’s Florida campus (895 patients, 976 controls), an Irish series (368 patients, 368 controls), and a Polish series (713 patients, 252 controls). Characteristics of subjects included in the study are summarized in Table 1 for each series. Patients were diagnosed with PD using standard criteria[13]. Controls were individuals free of PD or a related movement disorder at the time of examination. The Mayo Clinic Institutional Review Board approved the study and the review boards of the Mater Misericordiae University Hospital (Ireland), the Polish Academy of Sciences, the Medical University of Silesia, Jagiellonian University and the Central Hospital of the Ministry of Interior and Administration (Poland) received local IRB approvals, and all subjects provided written informed consent.

**Table 1:** Patient characteristics.

Series	N	Age	Age at onset	No. Male (%)
USA				
Cases	895	76.1±11.8 (35–104)	62.8±12.2 (16–94)	567 (63.4)
Controls	976	72.1±13.3 (25–98)		418 (42.8)
Ireland				
Cases	368	62.7±10.4 (32–87)	55.6±12.1 (18–87)	205 (55.7)
Controls	368	66.0±22.3 (17–97)		136 (37.0)
Poland				
Cases	713	67.0±12.1 (17–101)	56.8±11.9 (23–92)	433 (60.7)
Controls	252	57.7±15.7 (19–96)		143 (56.7)

The sample mean ± SD (minimum-maximum) is given for age and age at onset.

### Genetic analysis

DNA was extracted from peripheral blood monocytes according to a previously described protocol.[14] In the first stage of the study (screening stage), all the exons of genes *B3GALT1* (NM_020981.3, 2 exons), *STK39* (NM_013233, 18 exons), and *CERS6* (NM_001256126.1, 11 exons) were sequenced in 187 patients with familial late onset PD (from the US series). This series subset consists of 129 males (64%). These patients have a mean age of 80.4±8.1 (62–97) years old and a mean age at onset of 65.4±8.0 (51–83) years old. Non-synonymous variants were then validated (validation stage) by sequencing 376 control samples (168 males (45%), mean age 67.1±12.3 (29–88)) from the US. Bi-directional sequencing was performed as previously described[14]. In addition, the three insertions/deletions in exon 1 of *STK39* were genotyped by fragment sizing: PCR was performed using a fluorescently-labeled DNA primer, amplicons were run on an ABI 3730XL DNA sequencer (Applied Biosystems, Foster City, CA, USA), and reads were analyzed using GeneMapper 5 software (Life Technologies, Carlsbad, CA, USA). For the replication stage, all samples from the US, Irish, and Polish series (including the aforementioned 187 US PD patients and 376 US controls) were genotyped. *STK39* exon 1 insertion/deletion variants (del6: ss1570217805, ins3: ss1570217817, del21: ss1570217825) were genotyped using fragment sizing as previously described and the other identified variants (rs141683896, rs56031549, rs4496303, rs34110122) as well as GWAS tag rs2102808 were genotyped using TaqMan Allelic Discrimination Assays on an ABI 7900HT Fast Real-Time PCR system (Applied Biosystems, Foster City, CA, USA) and data was analyzed using Taqman Genotyper Software Version 1.3 (Applied Biosystems, Foster City, CA, USA). Primer sequences and amplification conditions are available upon request. Call rate for sequencing and genotyping was ≥98% at each stage.

### Statistical analysis

For stage 2 (validation), chi-square tests were used to compare the frequency of each variant between the 187 PD patients and 376 controls included in that stage. For stage 3 (replication), the association of each variant with PD was evaluated using a logistic regression model. ORs and 95% confidence intervals (CIs) were estimated, and each variant was considered under an additive model (i.e. effect of each additional minor allele). Additionally, to evaluate the effect of the coding single nucleotide polymorphisms (SNPs) on the GWAS association signal, we adjusted for each coding SNP individually and together in logistic regression models that included rs2102808 as a covariate (under an additive model). To test the combined effect of alleles, haplotype-based logistic regression analyses were performed on the variants with minor allele frequency (MAF) >1%, where only haplotypes occurring at a frequency of 1% or greater were considered. All regression models were adjusted for age, gender, and series (combined series only). Where indicated (P corr) P-values were corrected using the Bonferroni correction. P-values of 0.05 or lower were considered as statistically significant. All analyses were performed using PLINK v1.7 (http://pngu.mgh.harvard.edu/purcell/plink/)[15].

## Result

**Fig 1:**
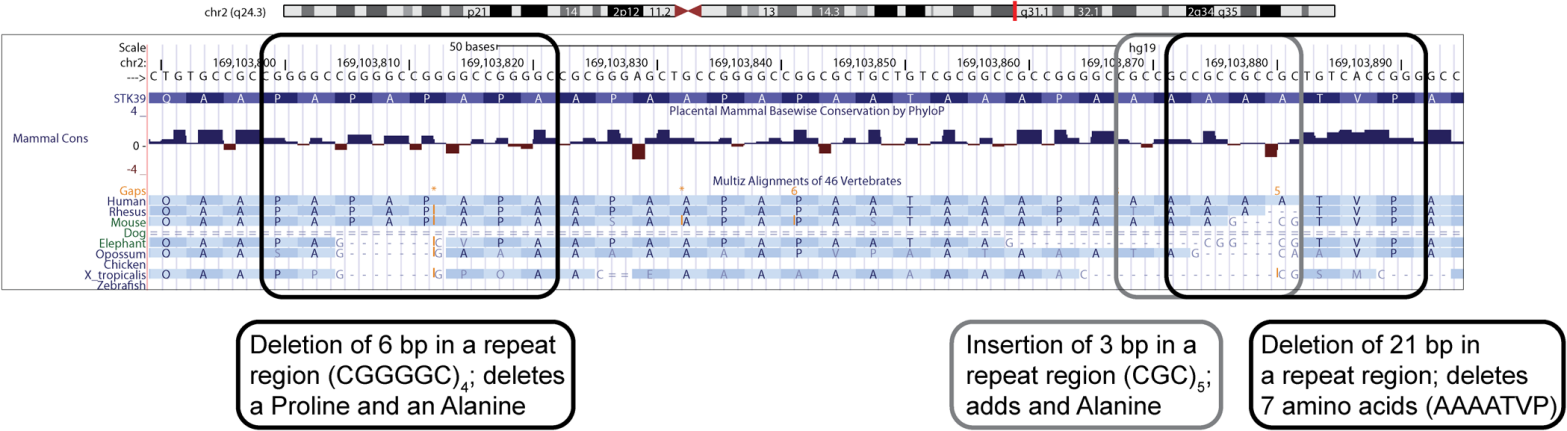
*STK39* exon 1 insertion/deletion variants. We detected three insertion/deletion (indels) variants in exon 1 of gene *STK39*. The indels are located in a proline/alanine rich protein domain called the PAPA box. The figure was created using the UCSC genome browser. (http://genome.ucsc.edu/)

We aimed to explain the PD GWAS signal at the chromosome 2q24.3 locus. In order to detect putative causal PD risk variants, we sequenced all 31 exons of genes *B3GALT1*, *STK39*, and *CERS6* in 187 PD cases from our US series. Upon sequencing of exon 1 of gene *STK39*, we identified a region rich in repeats and containing three in frame indels, an insertion of three base pairs, a deletion of six base pairs and a deletion of 21 base pairs (see Fig 1). We complemented our sequencing with fragment sizing to fully genotype these exon 1 variants. Following this screening, we identified 13 variants including four non-synonymous changes and three indels (Table 2). One non-synonymous SNP was located in *B3GALT1* (exon 2), three indels (exon 1) and one non-synonymous SNP (exon 11) were located in *STK39*, and two non-synonymous SNPs were located in *CERS6* (exon 1 and 5).

**Table 2:** Exonic variants detected by sequencing of 187 PD patients and validation in 376 controls.

Gene	Exon	Position on Chr2 (GRCh37)	rs/name	base change	AA change	MAF in Cases (%)	Allele counts in Cases	MAF in Controls (%)	Allele counts in Controls	OR (95% CI)	P
*B3GALT1*	1*	168675188	rs836722	C>T		19.84	65/303				
*B3GALT1*	1*	168675206	rs836723	C>T		19.84	65/303				
*B3GALT1*	2	168725616		T>C	L23L	0.27	1/371				
*B3GALT1*	2	168725741	rs141683896	A>T	E64D	0.53	2/372	0.28	2/724	1.95 (0.27–13.87)	0.50
*STK39*	11	168931636	rs56031549	C>T	A399T	1.10	4/360	0.41	3/723	2.68 (0.60–12.03)	0.18
*STK39*	3	169023879		G>A	N120N	0.27	1/371				
*STK39*	1	169103798	del6	CCGGGG>-	deletion PA	30.48	114/260	27.93	210/542	1.13 (0.86–1.49)	0.37
*STK39*	1	169103868	ins3	->CGC	insertion A	2.14	8/366	1.33	10/742	1.62 (0.63–4.14)	0.31
*STK39*	1	169103872	del21	GCCGCCGCCGCTGTCACCGGG>-	deletion AAAATVP	7.22	27/347	5.05	38/714	1.46 (0.88–2.43)	0.14
*CERS6*	1	169312974	rs4496303	G>A	A6T	0.81	3/367	0.95	7/731	0.85 (0.22–3.32)	0.82
*CERS6*	5	169547547	rs34110122	C>T	P157S	0.27	1/373	0.14	1/729	1.95 (0.12–31.33)	0.63
*CERS6*	7	169571519	rs116506340	C>T	G206G	0.80	3/371				
*CERS6*	8	169574432		G>A	L263L	0.27	1/371				

*Exon 1 of *B3GALT1* is non-coding, AA = amino acid, MAF = minor allele frequency, OR = Odds ratio, CI = confidence interval, P = P-value. Only non-synonymous and indel coding variants were genotyped in controls.

We prioritized the non-synonymous and indel variants as they are more likely to have a functional impact. To validate the non-synonymous variants identified, we sequenced (and genotyped through fragment sizing) 376 controls from our US series. Although none of the variants were statistically significant when comparing frequencies with the aforementioned 187 PD cases (Table 2), odds ratio estimates in this small patient-control group suggested that some may increase risk of PD. After evaluation of our statistical power to detect a significant association signal in our replication cohort, we decided to follow up on all non-synonymous variants.

In order to assess the role of our coding SNPs in PD risk at this locus, we genotyped all seven variants in all 1976 patients and 1596 controls from each of our three series (US, Irish, and Polish) and compared the association signal with GWAS locus tag SNP rs2102808 (chromosome 2 position 169117025 assembly GRCh37.p13). Fluorescent-based PCR fragment sizing of the indels allowed phasing of the three variants. Four alleles exist at the locus, they are: wt-wt-wt (70.3%), del6-wt-wt (20.9%), del6-wt-del21 (6.44%), and wt-ins3-wt (2.36%). The wt-ins3-wt allele sits on a haplotype with rs2102808 allele G (protective) and the del21 allele is more frequently transmitted with rs2102808 allele T (risk allele). The linkage disequilibrium between the four *STK39* SNPs and rs2102808 is presented in S1 Fig.

The results of the single variant association tests are shown in Table 3. The *STK39* ins3 variant consisting of an in frame insertion of three base pairs in a repeat region of exon 1 shows association with risk of PD in the US series (OR: 1.59, 95% CI: 1.01–2.51, P = 0.046), before correction for multiple testing, but this was not seen in the other series, including the large combined series (OR: 1.27, 95% CI: 0.91–1.75, P = 0.16). This is possibly due to population heterogeneity. The only SNP that was significantly associated with PD in the combined patient-control series was the GWAS tag SNP rs2102808 (OR: 1.23, 95% CI: 1.06–1.42, P = 0.005, P corr = 0.04). Additionally, in logistic regression analyses adjusting for the 2q24.3 locus coding variants, the GWAS association signal for rs2102808 was not altered (data not shown).

**Table 3:** Single variant associations with PD.

A. US series (895 patients, 976 controls)*							
Variant	MAF in cases (%)	Allele counts in cases	MAF in controls (%)	allele counts in controls	OR (95% CI)	P	P corr
rs141683896	0.17	3/1753	0.21	4/1930	0.86 (0.19–4.03)	0.85	1
rs56031549	1.29	23/1757	0.97	19/1933	1.43 (0.76–2.69)	0.27	1
del6	27.71	491/1281	26.52	516/1430	1.08 (0.93–1.25)	0.34	1
ins3	2.71	48/1726	1.8	35/1911	1.59 (1.01–2.51)	0.05	0.37
del21	5.64	100/1674	4.93	96/1850	1.17 (0.87–1.57)	0.29	1
rs2102808	14.74	263/1521	12.35	241/1711	1.3 (1.07–1.58)	0.009	0.07
rs4496303	1.19	21/1751	1.57	30/1882	0.67 (0.38–1.2)	0.18	1
rs34110122	0.11	2/1754	0.21	4/1924	0.44 (0.08–2.53)	0.36	1
B. Irish series (368 patients, 368 controls)*							
Variant	MAF in cases (%)	Allele counts in cases	MAF in controls (%)	allele counts in controls	OR (95% CI)	P	P corr
rs141683896	0.55	4/718	0.69	5/715	0.73 (0.19–2.8)	0.65	1
rs56031549	1.11	8/712	2.07	15/709	0.47 (0.19–1.16)	0.1	0.82
del6	26.67	192/528	24.72	179/545	1.08 (0.84–1.38)	0.54	1
ins3	2.64	19/701	2.9	21/703	0.83 (0.43–1.61)	0.59	1
del21	8.89	64/656	6.91	50/674	1.24 (0.84–1.82)	0.27	1
rs2102808	15.16	111/621	13.04	96/640	1.14 (0.85–1.53)	0.39	1
rs4496303	1.54	11/705	1.12	8/708	1.33 (0.52–3.4)	0.56	1
rs34110122	0.28	2/706	0.84	6/712	0.37 (0.07–1.88)	0.23	1
C. Polish series (713 patients, 252 controls)*							
Variant	MAF in cases (%)	Allele counts in cases	MAF in controls (%)	allele counts in controls	OR (95% CI)	P	P corr
rs141683896	0.14	2/1386	0.2	1/503	0.35 (0.03–4.2)	0.41	1
rs56031549	1.35	19/1391	0.41	2/488	3.6 (0.81–15.97)	0.09	0.73
del6	30.01	404/942	27.33	135/359	1.17 (0.92–1.48)	0.21	1
ins3	2.23	30/1316	2.43	12/482	0.98 (0.5–1.9)	0.94	1
del21	7.5	101/1245	8.1	40/454	0.96 (0.65–1.42)	0.85	1
rs2102808	14.89	207/1183	13.35	67/435	1.24 (0.89–1.71)	0.2	1
rs4496303	1.03	14/1342	1.81	9/489	0.51 (0.22–1.19)	0.12	0.95
rs34110122	0.22	3/1335	0	0/504	NA	1	1
D. Combined series (1976 patients, 1596 controls)*							
Variant	MAF in cases (%)	Allele counts in cases	MAF in controls (%)	allele counts in controls	OR (95% CI)	P	P corr
rs141683896	0.23	9/3857	0.32	10/3148	0.81 (0.31–2.12)	0.67	1
rs56031549	1.28	50/3860	1.14	36/3130	1.18 (0.75–1.86)	0.47	1
del6	28.32	1087/2751	26.23	830/2334	1.1 (0.98–1.22)	0.1	0.83
ins3	2.53	97/3743	2.15	68/3096	1.27 (0.91–1.75)	0.16	1
del21	6.9	265/3575	5.88	186/2978	1.13 (0.93–1.38)	0.22	1
rs2102808	14.87	581/3325	12.66	404/2786	1.23 (1.06–1.42)	0.005	0.04
rs4496303	1.2	46/3798	1.5	47/3079	0.76 (0.5–1.16)	0.2	1
rs34110122	0.18	7/3795	0.32	10/3148	0.64 (0.24–1.75)	0.39	1

MAF = minor allele frequency, OR = Odds ratio, CI = confidence interval, P = p-value, P corr = p-value with Bonferroni correction. ORs, 95% CIs, and P-values result from logistic regression models adjusted for age, gender, and series (combined series only).

*Numbers of samples with complete clinical information included in model.

We were interested in testing if haplotypes consisting of the GWAS SNP and the 2q24.3 coding SNPs carried increased risk to PD compared to single variants. Results for the three series are shown in Table 4. One haplotype defined by the rs2102808 minor allele (G>T) and a six base pair insertion in exon 1 of *STK39* (del6, CGGGGC>-) was significantly associated with PD in the combined series (OR = 1.35, 95% CI: 1.11–1.64, P = 0.003, P corr = 0.02). This particular haplotype is more significantly associated than rs2102808 by itself in the combined series (P corr = 0.04) and the OR suggests that it confers a slightly increased risk to PD than the GWAS variant (1.35 (1.11–1.64) compared to 1.23 (1.06–1.42)).

**Table 4:** Haplotypic associations with PD.

A. US series (895 patients, 976 controls)*					
Haplotype (rs56031549, del6, ins3, del21, rs2102808, rs4496303)	MAF in cases (%)	MAF in controls (%)	OR	P	P corr
111112	0.96	1.25	0.68 (0.34–1.34)	0.26	1
121221	5.3	4.62	1.17 (0.86–1.58)	0.31	1
121121	8.59	6.71	1.4 (1.09–1.8)	0.009	0.06
112111	2.64	1.74	1.63 (1.02–2.61)	0.04	0.25
122111	12.93	14.42	0.86 (0.71–1.04)	0.11	0.66
111111	69.58	71.27	0.91 (0.78–1.05)	0.18	1
B. Irish series (368 patients, 368 controls)*					
Haplotype (rs56031549, del6, ins3, del21, rs2102808, rs4496303)	MAF in cases (%)	MAF in controls (%)	OR	P	P corr
111112	1.19	0.68	1.86 (0.55–6.28)	0.32	1
121221	6.96	5.69	1.18 (0.77–1.81)	0.45	1
121121	6.99	5.58	1.23 (0.81–1.88)	0.34	1
112111	2.67	2.74	0.91 (0.47–1.78)	0.79	1
122111	10.05	10.86	0.92 (0.65–1.32)	0.66	1
111111	69.67	72.07	0.93 (0.73–1.18)	0.54	1
C. Polish series (713 patients, 252 controls)*					
Haplotype (rs56031549, del6, ins3, del21, rs2102808, rs4496303)	MAF in cases (%)	MAF in controls (%)	OR	P	P corr
111112	1.03	1.87	0.46 (0.2–1.09)	0.08	0.46
121221	6.85	7.4	0.97 (0.64–1.46)	0.87	1
121121	7.17	5.11	1.45 (0.9–2.34)	0.13	0.78
112111	2.2	2.22	1.07 (0.55–2.08)	0.84	1
122111	14.97	14.39	1.05 (0.78–1.41)	0.75	1
111111	67.78	69	0.93 (0.74–1.17)	0.53	1
D. Combined series (1976 patients, 1596 controls)*					
Haplotype (rs56031549, del6, ins3, del21, rs2102808, rs4496303)	MAF in cases (%)	MAF in controls (%)	OR	P	P corr
111112	1.03	1.26	0.74 (0.46–1.21)	0.23	1
121221	6.15	5.33	1.1 (0.9–1.35)	0.36	1
121121	7.86	6.27	1.35 (1.11–1.64)	0.003	0.02
112111	2.52	2.1	1.3 (0.93–1.81)	0.12	0.71
122111	13.19	13.66	0.92 (0.8–1.06)	0.27	1
111111	69.24	71.39	0.91 (0.82–1.02)	0.1	0.57

Haplotypes are encoded as 1 for major allele and 2 for minor allele see Table 2 for specific alleles, MAF = minor allele frequency, OR = Odds ratio, CI = confidence interval, P = p-value, P corr = P-value with Bonferroni correction. ORs, 95% CIs, and P-values result from haplotype-based logistic regression analysis.

*Numbers of samples with complete clinical information included in model.

## Discussion

Our screening at the PD GWAS locus at 2q24.3 identified a risk haplotype defined by rs2102808 allele T as well as a six base pair deletion in exon 1 of the *STK39* gene. This haplotype is associated with an increased risk of PD (p = 0.003, OR = 1.35) with an estimated effect size that is greater than the effect observed when these alleles are tested independently (OR = 1.1 [del6] and 1.23 [rs2102808]). Given that the strength of the association is greater for this haplotype than for each single allele individually (p = 0.10 and 0.005), it is possible to suspect a contribution of the del6, or an untested variant in LD with it, to PD, although with a small effect size.

Our screening did not identify a single common coding variant responsible for the locus association signal which suggests at least two scenarios: 1) the causal risk factor at the locus consists of several different variants of low frequency and our sample size is too small to detect individual effects, or 2) the causal variant is located outside of the coding region. In the latter case, a screening of the non-coding regions might identify variants located in regulatory elements such as promoter and enhancers that modulates gene expression levels. Of interest for the study of PD, BioGPS reports *STK39* mRNA levels to be greatest in brain regions compared to other tissue tested with the affymetrix expression microarray U133, whereas *CERS6* mRNA levels are higher in dendritic cells and in the pineal gland and *B3GALT1* is expressed ubiquitously[16]. Protein STK39 is a kinase involved in the phosphorylation and activation of Na^+^-K^+^-Cl^-^ co-transporters. These transporters are implicated in the neuronal depolarizing response led by GABA and glycine neurotransmitters via changes in the intracellular concentration of Cl^-^. [17] *STK39* knockout mice have been shown to have higher nociceptive threshold, impaired motor function and increased anxiety [18].

Although no conclusions can be drawn as to the location of the causal variants based on this particular study, the *STK39* exon 1 is an interesting candidate region in the search for regulatory variants, as it contains many repeat elements. The exon encodes a proline/alanine rich region (amino acids 12 to 53) called the PAPA box for which the precise function is still unknown[17]. The PAPA box is designated as an active promoter region and includes a CTCF binding site based on ENCODE ChIP-seq data[19]. CTCF is a ubiquitously expressed protein which functions as transcriptional repressor, activator or an insulator blocking enhancer activity and thus influencing gene expression[20]. We identified three indels located in the PAPA box but none of these variants were significantly associated with PD risk. The variant located on the associated haplotype is a two amino acid deletion with a minor allele frequency of ~26%. The variant is located in a repeat motif (unit: CGGGGC) with the major allele being five repeat units and the minor four.

Resolving the underlying genetic variation at each GWAS loci that is associated with disease susceptibility is critical to our understanding of not only the clinical relevance but also the disease mechanisms. This goal is challenging and even for the loci that overlap with known familial PD genes (e.g. *SNCA* and *LRRK2*), the functional associated variants accounting for the GWAS signal have not yet been identified. The exonic portion of the *LRRK2* gene, recognized as the most common genetic cause of both familial and sporadic PD, has been extensively studied by our group [21]. Although low penetrant variants have been identified and confirmed to modestly increase or decrease disease risk, these associations do not explain the GWAS signal at the *LRRK2* locus [22]. This most likely reflects the presence of functional/regulatory variants located outside of the coding region accounting for the association signal. This is also the case for the *SNCA* gene with no common coding variation observed, and may be a common phenomenon for a number of the other GWAS nominated loci. If this is true, additional genetic sequencing studies with increased sample size and a focus extended to non-coding regulatory regions will be needed to pinpoint to the precise variants responsible for the association signal at locus Chromosome 2q24.3.

## Supporting Information

S1 Fig Linkage disequilibrium (LD) between chromosome 2q24.3 variants. Left panel (A) shows the D’ values and right panel the r2 values. The LD was calculated in the US control samples and the figure was created using Haploview (Barrett JC, Fry B, Maller J, Daly MJ. Haploview: analysis and visualization of LD and haplotype maps. Bioinformatics. 2005 Jan 15). A. Numbers on the squares represent D’(x100) between two variants, no number mean D’ = 1. A white square represents LOD scores less than 2 and D’ less than 1 (low LD), a light blue square represents D’ = 1 but LOD score less than 2. Shades of pink squares represent D’ less than 1 and LOD score more than 2 and bright red squares show variant in LD, D’ = 1 and LOD score more than 2. B. Shades of grey squares represent the correlation between variants expressed as r2 (x100). Del6, del 21 and rs2102808 are in high LD but have very different minor allele frequencies, hence the high D’ and low r2.
(PDF)
