# Supplementary material for: Exonic Re-Sequencing of the Chromosome 2q24.3 Parkinson’s Disease Locus
Source: PLoS One. 2015 Jun 19;10(6):e0128586. doi: 10.1371/journal.pone.0128586 (PMC4474914; doi:10.1371/journal.pone.0128586)
Supplement: S1 Fig — Left panel (A) shows the D’ values and right panel the r2 values. The LD was calculated in the US control samples and the figure was created using Haploview (Barrett JC, Fry B, Maller J, Daly MJ. Haploview: analysis and visualization of LD and haplotype maps. Bioinformatics. 2005 Jan 15). A. Numbers on the squares represent D’(x100) between two variants, no number mean D’ = 1. A white square represents LOD scores less than 2 and D’ less than 1 (low LD), a light blue square represents D’ = 1 but LOD score less than 2. Shades of pink squares represent D’ less than 1 and LOD score more than 2 and bright red squares show variant in LD, D’ = 1 and LOD score more than 2. B. Shades of grey squares represent the correlation between variants expressed as r2 (x100). Del6, del 21 and rs2102808 are in high LD but have very different minor allele frequencies, hence the high D’ and low r2. (PDF) [file pone.0128586.s001.pdf]

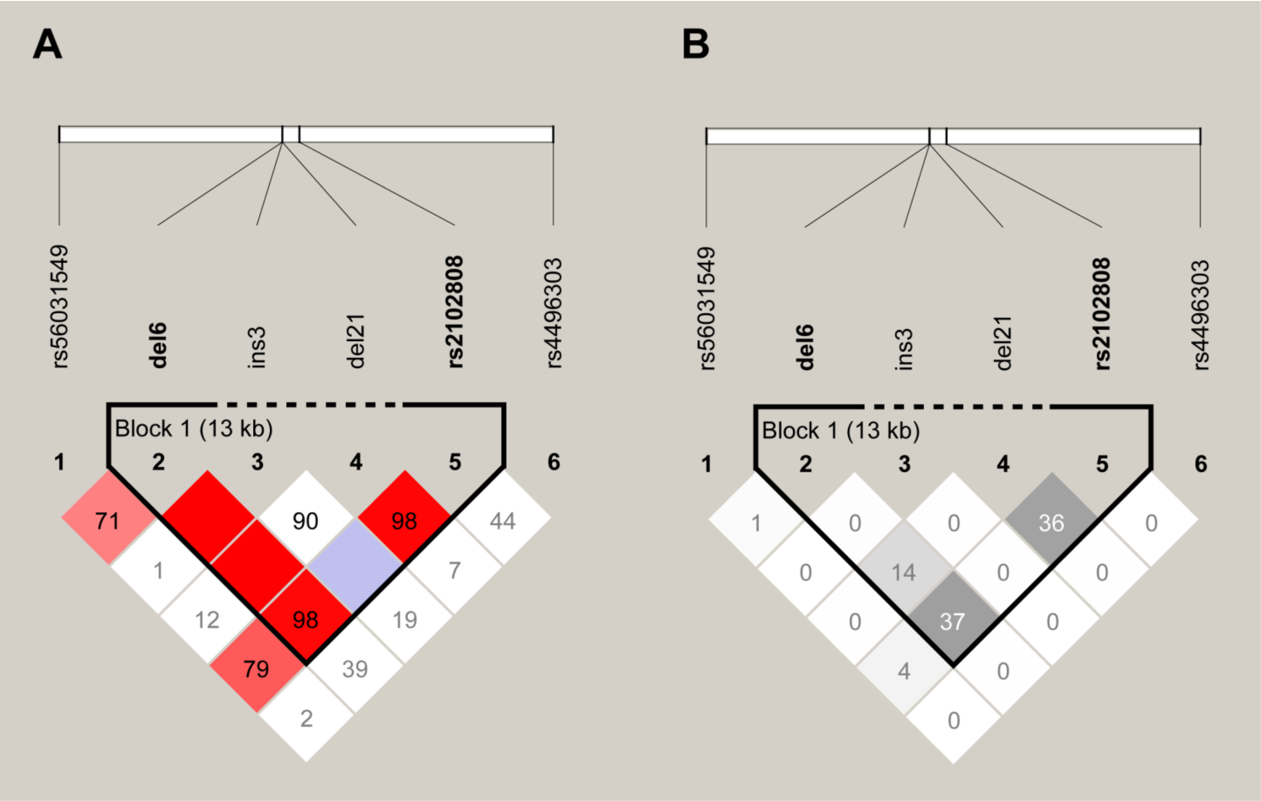

S1 Fig. Linkage disequilibrium (LD) between chromosome 2q24.3 variants

The left panel (A) shows the D’ values and the right panel the r^2 values. The LD was calculated in the US control samples and the figure was created using Haploview (Barrett JC, Fry B, Maller J, Daly MJ. Haploview: analysis and visualization of LD and haplotype maps. Bioinformatics. 2005 Jan 15). A. Numbers on the squares represent D’(x100) between two variants, no number mean D’=1. A white square represents LOD scores less than 2 and D’ less than 1 (low LD), a light blue square represents D’=1 but LOD score less than 2. Shades of pink squares represent D’ less than 1 and LOD score more than 2 and bright red squares show variant in LD, D’=1 and LOD score more than 2. B. Shades of grey squares represent the correlation between variants expressed as r^2 (x100). Del6, del 21 and rs2102808 are in high LD but have very different minor allele frequencies, hence the high D’ and low r^2.
